# Flu on the Brain: Identification of Highly Pathogenic Influenza in the Brains of Wild Carnivores in The Netherlands

**DOI:** 10.3390/pathogens12091111

**Published:** 2023-08-30

**Authors:** Kristian T. Barry, Michelle D. Tate

**Affiliations:** 1 Centre for Innate Immunity and Infectious Diseases, Hudson Institute of Medical Research, Clayton, VIC 3168, Australia; kristian.barry@hudson.org.au; 2 Department of Molecular and Translational Sciences, Monash University, Clayton, VIC 3168, Australia

Highly pathogenic avian influenza (HPAI) viruses circulate in wild birds and can infect domestic poultry. H5NX 2.3.4.4b is a clade of HPAI responsible for major avian epidemics seen in Europe in the 2016–2017 and 2020–2021 seasons, with the 2021–2022 season being the largest so far in terms of geographic spread and number of detections in non-avian animals [1]. While in recent months poultry infections have declined, HPAI H5NX continues to circulate in wild birds, and HPAI H5N1 genotypes have infected several mammal species in Europe, the Americas, and Asia [2]. Indeed, HPAI H5N1 detected in red foxes in The Netherlands have been found to carry a PB2-E627K mutation that increases viral replication in mammalian cells [3]. Furthermore, HPAI H5N1 was identified to have a neurotropism in these foxes, causing infection in the brain.

In the current study [4], Vreman et al. build on this previous report by expanding their testing to other carnivorous species within The Netherlands for surveillance, with a particular interest in those showing neurological signs. The authors found that 60% of carnivores with neurological signs tested positive for HPAI H5N1 IAV, demonstrating that HPAI H5N1 was highly capable of infecting several carnivore species (including foxes, polecats, otters, and badgers), with the virus detected in the brain despite low or no virus detected in nasal or anal swabs. This was significant, as the testing of mammals routinely only tests nasal and anal swabs, meaning that routine surveillance of mammals may not fully identify infected cases. While most of the carnivores carried the PB2-E627K mutation that allows for easier infection of mammalian cells, full genome sequencing of the virus identified that each infection came from distinct HPAI H5N1 strains, making it unlikely that there had been any mammal-to-mammal transmission and suggesting the carnivores may have acquired the virus from infected birds. 

The researchers also investigated the route of transmission to the carnivores. Given the scavenging nature of the carnivores studied, particularly foxes and polecats, the authors hypothesised that the carnivores may have become infected by consuming sick or dead infected birds, as had been seen in previous studies. Indeed, one former study in experimentally infected cats identified that consuming HPAI H5N1-infected chicks caused brain lesions similar to those in the present study [5]. Infection in cats was linked to viral lesions in the nervous plexus of the small intestines; however, the researchers were unable to identify the infection of intestinal nerves in wild carnivores, although this may be explained through the autolysis of tissue prior to collection, given the nature of the sample collection from carcasses. The investigation of live infected animals may elucidate infection pathways in future studies.

While Vreman et al. only identified HPAI infections in wild carnivorous mammals that do not often come into contact with humans, other recent studies have identified that HPAI H5N1 is also able to infect domestic cats in France and Poland, which have a much higher chance of passing the virus to humans [6,7]. All cats also carried the PB2-E627K mutation alongside other mutations related to mammalian adaptation. Of note, infected cats also presented with neurological signs, and the virus was present in the brain, in accordance with the current study on wild carnivores in The Netherlands. Taken together, as stressed by Vreman et al., surveillance of carnivorous mammals that show neurological signs of disease, particularly testing brain tissue, is paramount to monitoring HPAI H5N1 infections and ensuring spread to humans is kept to a minimum, particularly as HPAI IAV outbreaks continue to break infection records every year.
